# Fibrous dysplasia: rare manifestation in the temporal bone

**DOI:** 10.1016/j.bjorl.2020.05.027

**Published:** 2020-07-21

**Authors:** Thaís de Carvalho Pontes-Madruga, Halana Valéria Carneiro Filgueiras, Daniel Marcus San da Silva, Leonardo Sales da Silva, José Ricardo Gurgel Testa

**Affiliations:** Universidade Federal de São Paulo (UNIFESP), Escola Paulista de Medicina, São Paulo, SP, Brazil

**Keywords:** Temporal bone, Craniofacial dysostosis, Cholesteatoma

## Abstract

**Introduction:**

Fibrous dysplasia is a benign disorder, in which normal bone is replaced by fibrosis and immature bone trabeculae, showing a similar distribution between the genders, and being more prevalent in the earlier decades of life. Fibrous dysplasia of the temporal bone is a rare condition, and there is no consensus as to whether it is more common in monostotic or polyostotic forms. External auditory meatus stenosis and conductive dysacusis are the most common manifestations, with cholesteatoma being a common complication, whereas the involvement of the otic capsule is an unusual one. Surgical treatment is indicated to control pain or dysacusis, otorrhea, cholesteatoma, and deformity.

**Objectives:**

To describe the clinical experience of a tertiary referral hospital with cases of fibrous dysplasia of the temporal bone.

**Methods:**

Sampling of patients diagnosed with fibrous dysplasia of the temporal bone, confirmed by tomography, treated at the pediatric otology and otorhinolaryngology outpatient clinics, between 2015 and 2018. The assessed variables were age, gender, laterality, external auditory meatus stenosis, deformity, hearing loss, presence of secondary cholesteatoma of the external auditory meatus, lesion extension and management.

**Results:**

Five patients were included, four females and one male, with age ranging from 13 to 34 years. Three had the polyostotic form and two the monostotic form of fibrous dysplasia of the temporal bone. Four patients had local deformity and external auditory meatus stenosis, two of which progressed to cholesteatoma. All patients showed some degree of hearing impairment. All had preserved otic capsule at the tomography. Two patients are currently undergoing clinical observation; two were submitted to tympanomastoidectomy due to secondary cholesteatoma; one was submitted to lesion resection, aiming to control the dysacusis progression.

**Conclusion:**

Five cases of fibrous dysplasia of the temporal bone were described, a rare disorder of which the otologist should be aware.

## Introduction

Fibrous dysplasia is a benign, slowly progressive disorder of unknown origin, in which normal bone is replaced by disorganized and immature fibrous and trabecular bone tissue.^1^, ^2^, ^3^ This tissue tends to expand and thin out the bone cortex.^4^

Its prevalence is around 1–2 cases for every 30,000 births, with a similar distribution between males and females, and is more common in the first decades of life. It often affects craniofacial bones, long bones and ribs.^1^

It has three forms: monostotic (affects only one bone), non-syndromic polyostotic (affects more than one bone) and McCune-Albright Syndrome, defined by the presence of fibrous dysplasia associated with endocrinological disorder and café-au-lait spots.^1^, ^3^, ^4^, ^5^ The craniofacial skeletal involvement occurs in about 50% of the polyostotic forms, 27% of the monostotic forms and in 90% of McCune-Albright syndrome cases.^6^

The diagnosis is based on a combination of clinical, radiological, histological and genetic findings. The clinical examination of craniofacial skeletal dysplasia shows an increase in local volume with facial asymmetry, with the maxillary bone being the most often affected, followed by the mandibular, frontal, sphenoid, ethmoid, parietal, temporal and occipital bones.^7^ Although the histopathological exam is considered the gold standard, computed tomography is the most accurate imaging exam for the diagnosis of bone fibrous dysplasia, as well as for treatment planning and follow-up,^8^, ^9^ and the biopsy can be avoided in typical cases of stable disease.^2^

Temporal bone involvement is uncommon and usually unilateral,^2^, ^3^ with an estimated 11% −12% of cases of craniofacial bone fibrous dysplasia.^7^

## Objective

The aim of this study is to describe the clinical experience of a tertiary referral hospital with cases of Fibrous Dysplasia of the Temporal Bone (FDTB).

## Method

This was a descriptive, observational study carried out in the pediatric otology and otorhinolaryngology outpatient clinics of a tertiary hospital, between 2015 and 2018.

Patients diagnosed with fibrous dysplasia of the temporal bone, of any laterality, confirmed by temporal bone tomography were included in the study. Patients with an uncertain diagnosis, without the classic ground-glass aspect on temporal bone tomography were excluded.

The patients were chosen according to their presentation at the pediatric otology and otorhinolaryngology outpatient clinics. For each patient, a form was filled out with the variables: age, gender, laterality of FDTB, stenosis of the external auditory meatus, bulging of the temporal region, hearing loss, otorrhea, secondary cholesteatoma, extension to the temporal bone squamous portion, mastoid, labyrinth and petrous portion and to the adjacent bones in the computed tomography, adopted conduct (conservative or surgical) and evolution.

The patients’ auditory investigation was carried out through pure tone audiometry, between 250 Hz and 8000 Hz. Clinically significant hearing losses were defined by obtaining the mean tone thresholds of PTA (pure tone average) at 500 Hz, 1000 Hz, 2000 Hz and 4000 Hz. For the degree of hearing loss, the World Health Organization (WHO, 2014) classification was used: mild (26−40 dB), moderate (41−60 dB), severe (61−80 dB) and profound (>80 dB). The classification of Silman and Silverman (1997) was used to define the type of hearing loss, using the difference between the PTA of the air conduction and the PTA of the bone conduction at 500 Hz, 1000 Hz and 2000 Hz: conductive, with bone conduction thresholds lower than or equal to 15 dB and air conduction thresholds greater than 25 dB, with a bone-air gap greater than or equal to 15 dB; sensorineural, with bone conduction thresholds greater than 15 dB and air conduction thresholds greater than 25 dB, with bone-air gap of up to 10 dB; mixed, with bone conduction thresholds greater than 15 dB and air conduction thresholds greater than 25 dB, with bone-air gap greater than or equal to 15 dB.^10^

The assessment of the disease extension was performed by obtaining computed tomography scans of the temporal bones, evaluated at the axial and coronal views.

According to the norms of Resolution 466/12 of the National Health Council / MS for Research Involving Human Beings, this research project was submitted to the Research Ethics Committee and was approved under number CAAE 97625718.8.0000.5505.

## Results

Five patients with FDTB, who were being followed at the outpatient clinic during the study period, were identified.

### Demographic data

Four patients were females and one was male. The age ranged from 13 to 34 years, with a median of 19 years. Four patients had FDTB on the left and one on the right side.

### Extension of lesions at the tomography

**Figure 1:**
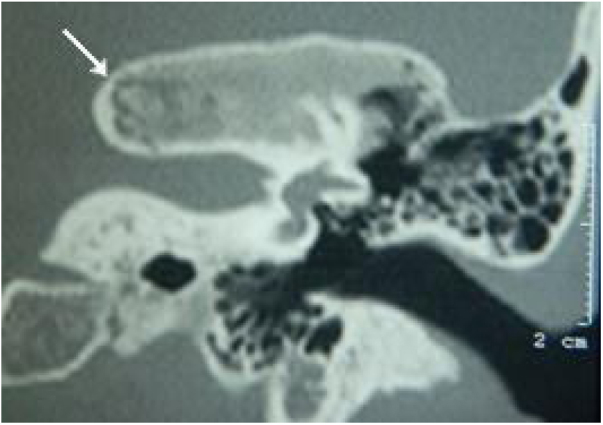
Tomography of the temporal bones, coronal section, left side. The arrow indicates monostotic fibrous dysplasia restricted to the petrous portion.

**Figure 2:**
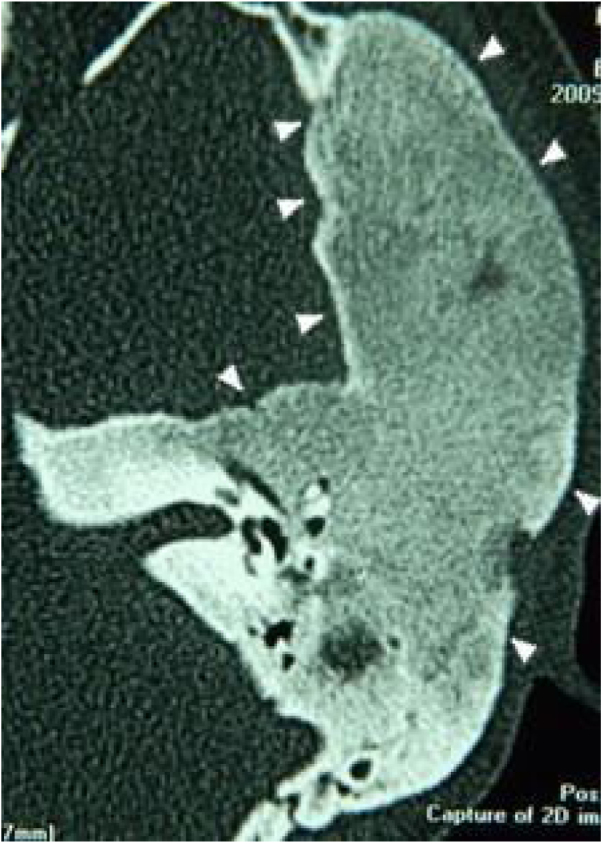
Tomography of the temporal bones, axial section, left side. Arrowheads: Monostotic fibrous dysplasia, extending to all portions of the temporal bone and restricted to their sutures.

All patients had the disease confirmed based on the ground-glass aspect of the temporal bone at the computed tomography. Two patients had the monostotic form, one with a small focus of FDTB restricted to the postero-superior region to the labyrinth of the petrous portion (Fig. 1) and the another with disseminated FDTB, from the most posterior region of the mastoid to the zygomatic arch and superiorly throughout the squamous portion, however restricted to the temporal bone sutures (Fig. 2).

**Figure 3:**
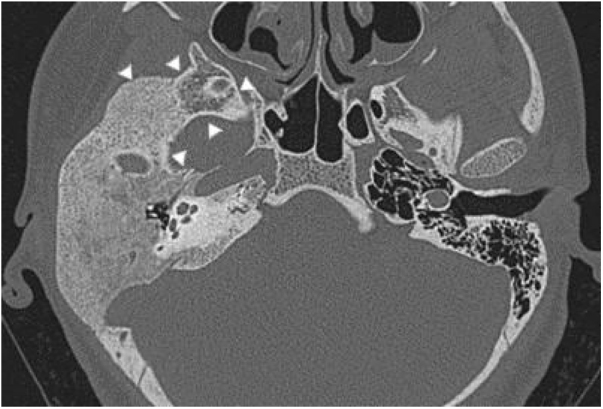
Tomography of the temporal bones, axial section. Polyostotic fibrous dysplasia of the temporal bone on the right. The arrowheads indicate extension to the larger sphenoid wing.

Three patients presented the polyostotic form of the disease, with extension to the larger wing of the sphenoid bone and one of them with involvement of the parietal portion, adjacent to the squamous portion (Fig. 3).

**Figure 4:**
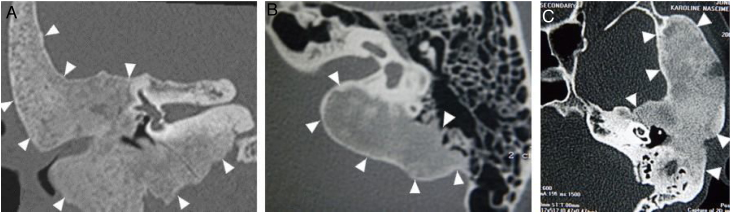
Three patients submitted to temporal bone tomography, with the arrowheads showing variable extension of the fibrous dysplasia, sparing the optical capsule. (A) Coronal view, right side. (B) Axial view, left side. (C) Axial view, left side.

The narrowing of the tympanic cleft by the expansive lesion is observed on CT scans, with the exception of one patient with a lesion restricted to the petrous portion. The ground-glass lesion touches the otic capsule in all patients, even surrounding it, but the dense bone tissue surrounding the labyrinth is preserved in all patients (Fig. 4).

### Clinical manifestations

**Figure 5:**
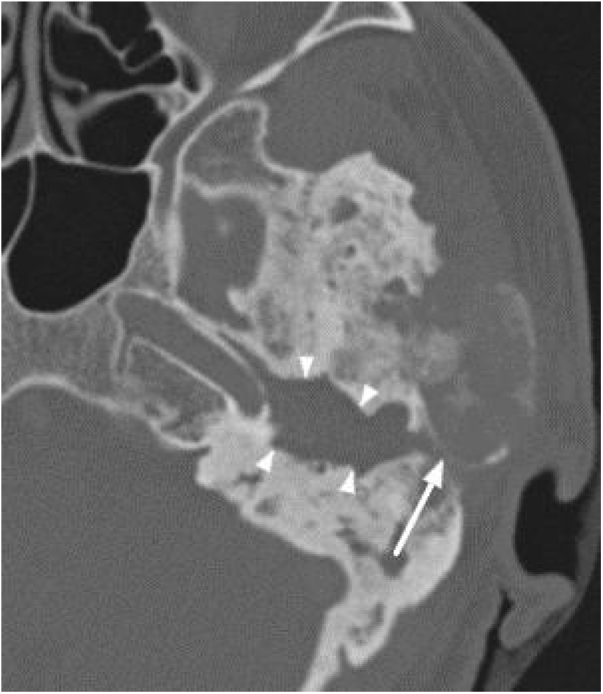
Tomography of temporal bones, axial view, left side. The arrow shows stenosis of the external auditory meatus and the arrow heads indicate erosion and filling of soft tissue contents - secondary cholesteatoma.

**Figure 6:**
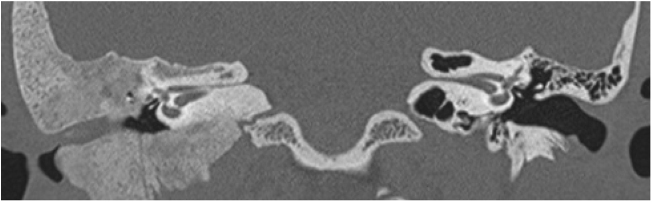
Tomography of temporal bones, coronal view. Presence of stenosis in the right external auditory meatus, without erosion.

Four patients had swelling of the affected temporal region and stenosis of the external auditory meatus at the beginning of the follow-up, of which two of them developed otorrhea and cholesteatoma secondary to FDTB (Fig. 5), whereas one of them had two episodes of otalgia and otorrhagia, but without debris or erosions (Fig. 6). One patient had a completely normal otoscopy and no signs of cranial deformities, with the lesion restricted to the petrous portion. One patient complained of temporoparietal headache in the area of the lesion.

### Audiological findings

**Figure 7:**
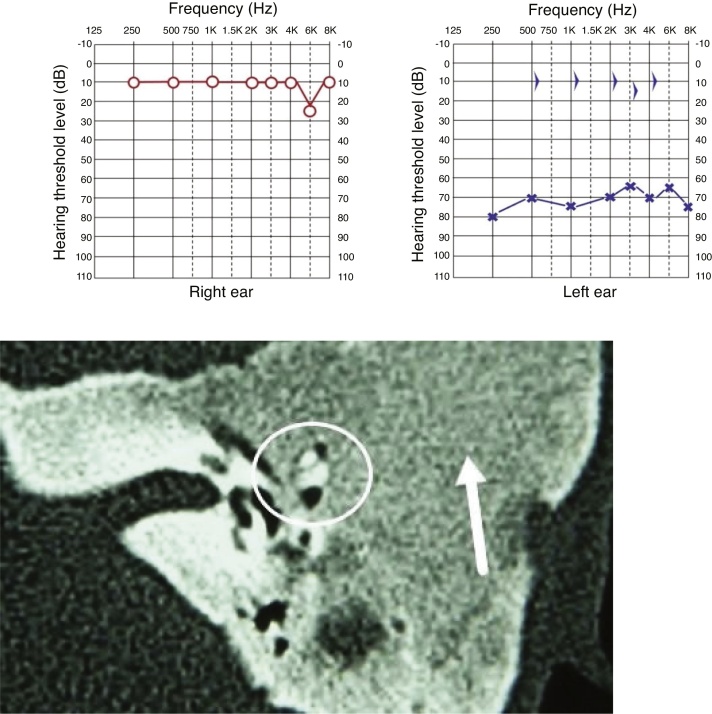
Pure tone audiometry with severe conductive hearing loss. Tomography of temporal bones in the axial view, left side. The circle shows a reduction in the tympanic cleft and the arrow shows ground-glass lesion affecting the squamous and mastoid portion.

**Figure 8:**
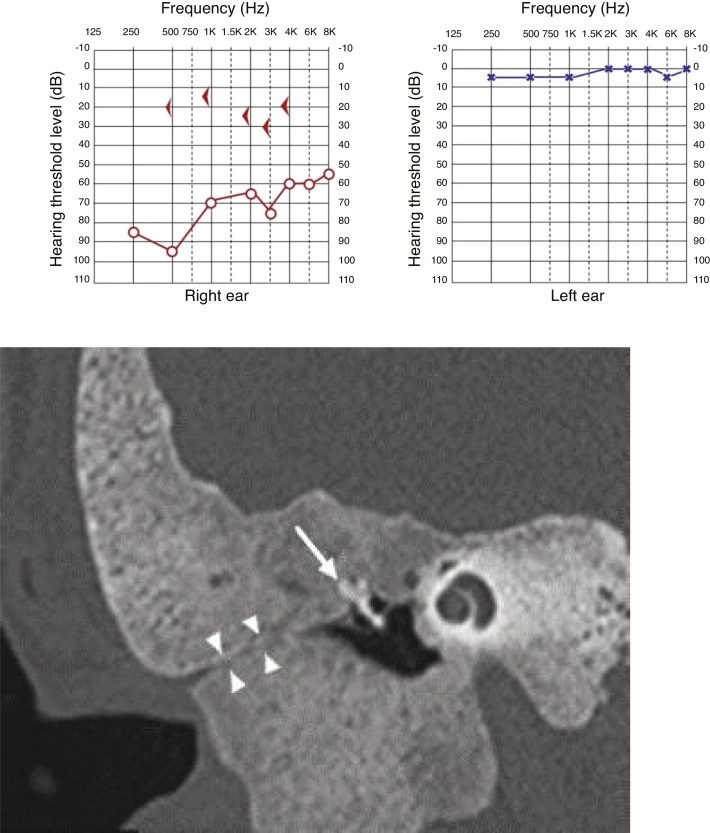
Pure tone audiometry showing severe conductive hearing loss and the presence of Carhart’s notch. Tomography of temporal bones, coronal view, right side. The arrow heads show stenosis of the external auditory meatus and the arrow shows ossicular chain with fixation.

All cases showed some degree of hearing impairment. The patient who had a focal lesion in the petrous portion had a bone-air gap of 5 dB, associated with dizziness. The other patients, with a greater extension of FDTB, had moderate to severe conductive or mixed hearing losses (Figure 7, Figure 8).

### Follow-up and treatment

During follow-up, an observation conduct was indicated for two patients. One of them, at age 30, had focal FDTB in the petrous portion on the left and associated mild hearing loss, without stenosis of the meatus or craniofacial deformity. The other, aged 13, had a polyostotic lesion that extended throughout the temporal bone, severe stable hearing loss, bulging of the temporal region and stenosis of the external auditory meatus, but no otorrhea or signs of complications due to cholesteatoma.

**Figure 9:**
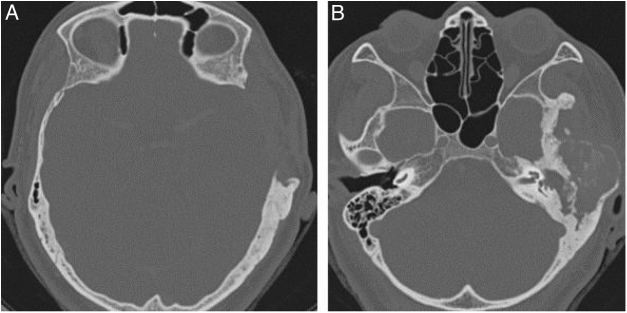
Tomography of temporal bones, axial views. (A) Partial resection of the squamous and parietal portions on the left. (B) Evolution with cholesteatoma in the mastoid and ipsilateral tympanic portions.

Three patients underwent surgery. Of the three, one had a surgical indication due to cholesteatoma secondary to stenosis of the external auditory meatus, presenting otorrhea and undergoing radical mastoidectomy, maintaining sporadic cleaning of the surgical cavity. Another patient underwent partial resection of the squamous portion of the temporal bone apart from the parietal bone by a neurosurgery team, with the intention of controlling headache complaints and swelling in this region; however, she developed otorrhea and cholesteatoma was diagnosed in the outer and middle ear regions, and a radical mastoidectomy was performed by the otology team. She has maintained the surgical cavity with a wide meatoplasty, with no signs of cholesteatoma recurrence (Fig. 9).

**Figure 10:**
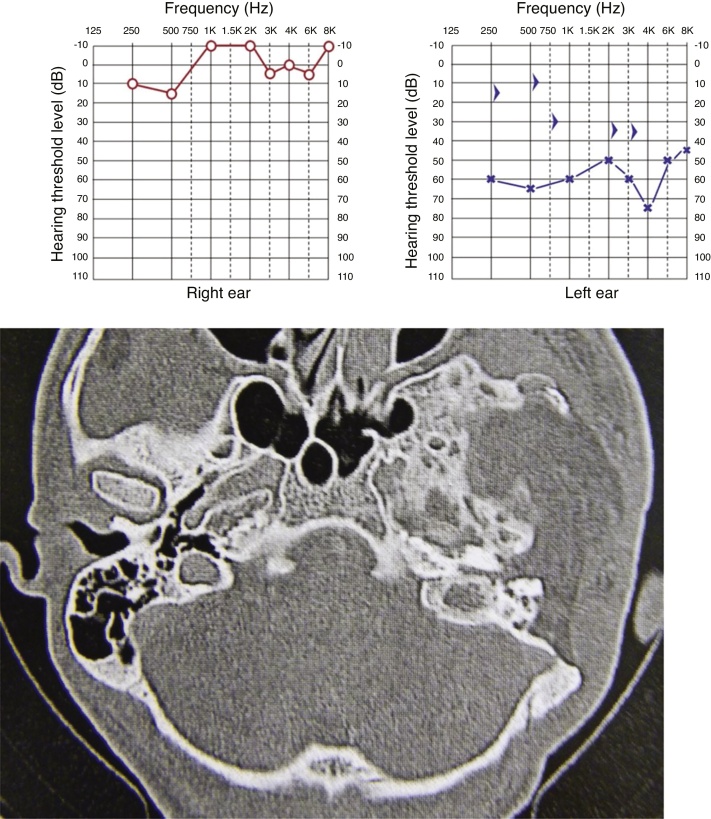
Pure tone audiometry showing moderate hearing loss. Tomography of temporal bones, axial view, showing resection of a dysplastic lesion of the temporal bone on the left, with a small remnant in the petrous portion and exclusion of the external auditory meatus.

The last patient had moderate mixed hearing loss and stenosis of the external auditory meatus. Extensive lesion resection was performed, excluding the external auditory meatus due to disease progression. The patient maintained a moderate mixed hearing loss after surgery and is not interested in rehabilitation with bone-anchored hearing aids (Fig. 10).

The anatomopathological reports of the surgically-resected lesions reported proliferation of spindle fibroblastic cells without atypia and new trabecular bone formation or mineralized component with a psammomatous aspect, with no evidence of malignancy. Table 1 summarizes the clinical and demographic characteristics of patients with FDTB.

**Table 1:** Demographic data and clinical characteristics of patients with fibrous dysplasia of the temporal bone.

	Median	Range
Age (years)	19	13 − 34
	**n**	**%**
Gender		
Male	1	20
Female	4	80
Laterality		
Right	1	20
Left	4	80
Type of fibrous dysplasia		
Monostotic	2	40
Polyostotic	3	60
Stenosis of the external auditory meatus	4	80
Esthetic deformity	4	80
Headache	1	20
Hearing loss	5	100
Conductive	4	80
Sensorineural	0	0
Mixed	1	20
Mild	1	20
Moderate	1	20
Severe	3	60
Otorrhea	2	40
Secondary cholesteatoma	2	40
Treatment		
Clinical observation	2	40
Open mastoidectomy	2	40
Lesion resection	1	20

## Discussion

FDTB is a rare entity, although its prevalence can be underestimated due to a percentage of asymptomatic cases.^1^ This is a disease that typically appears in the first decades of life, with a mean age at the diagnosis ranging from 16 to 25 years,^1^, ^11^, ^12^ a characteristic compatible with the findings of the present study.

There was a predominance of females, which also occurs in other published samples, but less significantly, of around 55%–59%.^1^, ^11^

There is no consensus in the literature regarding the most prevalent form of fibrous dysplasia affecting the temporal bone.^1^, ^5^, ^6^ Megerian et al. identified 70% of patients with the monostotic form of the disease.^12^ On the other hand, Frisch et al. describe a proportion of 89% of patients with the polyostotic and 11% with the monostotic form, with 24% of occurrence of McCune-Albright syndrome^1^; Boyce et al. reported an occurrence of 94% of the polyostotic form and 6% of the monostotic form.^11^ This finding can be justified by a conceptual disagreement: while Megerian et al. considered single lesions that extend across different skull bones as monostotic disease, the other authors classify such cases as polyostotic disease.^1^ The present study describes two monostotic and three polyostotic forms.

Due to the slow evolution, patients are often asymptomatic.^3^ In this study, all patients had hearing disorders, including the patient with a smaller and more focal lesion. The most common manifestations are stenosis of the external auditory meatus, with conductive hearing loss,^2^ present in 80% of the patients in this study. One patient had only mild conductive hearing loss, with no meatal involvement due to the focal lesion in the petrous portion. Progressive stenosis leads to the incarceration of epithelial cells, resulting in the formation of secondary cholesteatoma,^13^ and may show an extension to the middle ear, with erosion or fixation of the ossicular chain,^2^ which happened in two patients of this sample.

Sensorineural hearing loss may result from the involvement of the otic capsule or from complications of cholesteatoma.^2^ Boyce et al. identified a positive correlation between sensorineural hearing loss and the length of the internal auditory meatus, suggesting a lesion mechanism by neuronal stretching in these patients.^11^ All patients in this study had a preserved optical capsule at the tomography, even those who had a ground-glass lesion around its entire circumference. In previous case reports, inner ear involvement by FDTB was alsonot common.^2^

Asymptomatic or oligosymptomatic patients can be followed with clinical observation.^2^, ^13^ Surgical treatment is recommended in cases of stenosis of the external auditory meatus with recurrent infections or secondary cholesteatoma, local pain, conductive hearing loss, craniofacial deformity, pathological fracture, nerve decompression and in cases where anatomopathological examination is necessary for differential diagnosis with malignant diseases.^2^, ^14^ In this study, two patients underwent resection of FDTB due to complications from cholesteatoma, one of which was submitted to a previous partial resection, indicated for relief of severe local pain and cranial deformity. One patient underwent surgery to stop the progression of hearing loss.

Frisch et al., in 2014, reported 66 cases of FDTB and described that the most adequate surgical treatment modality was tympanomastoidectomy, followed by incisional biopsy, canaloplasty, debulking and partial resections for cosmetic purposes.^1^ In this study, two patients were submitted to tympanomastoidectomy with meatoplasty to partially reduce the lesion and secondary cholesteatoma. One of these patients had been previously undergone *en bloc* resection of the squamous and parietal portions of the lesion for headache control, an indication that was not found in the cases described in the literature. Another information in our sample that was different from the literature is that a patient was submitted to a temporalectomy in an attempt to completely remove the lesion. We did not find in our references any information regarding temporalectomy in patients with FDTB.

## Conclusion

The rare occurrence of FDTB makes it difficult to better characterize its clinical manifestations and define empirical approaches. Our sample showed clinical manifestations similar to those in the literature, with a predominance of conductive hearing loss and stenosis of the EAM, in addition to the appearance of secondary cholesteatoma. There was also agreement regarding the predominance of young individuals and women. This is a rare disorder, of which the otologist must be aware.

## Conflicts of interest

The authors declare no conflicts of interest.
